# Complete Genome Sequence of a Novel Thermoacidophilic and Facultatively Anaerobic Crenarchaeon, *Stygiolobus* sp. Strain KN-1

**DOI:** 10.1128/MRA.00581-21

**Published:** 2021-09-30

**Authors:** Koichi Nakamura, Norio Kurosawa, Hiroyuki D. Sakai

**Affiliations:** a Department of Environmental Engineering for Symbiosis, Graduate School of Science and Engineering, Soka University, Hachioji, Tokyo, Japan; b Department of Science and Engineering for Sustainable Innovation, Faculty of Science and Engineering, Soka University, Hachioji, Tokyo, Japan; Indiana University, Bloomington

## Abstract

The complete genome sequence of the thermoacidophilic crenarchaeon *Stygiolobus* sp. strain KN-1 was determined and annotated. The genome was 2,958,410 bp in size, with a GC content of 40.1%. It contained 2,973 coding sequences, a single copy of the 16S-23S rRNA operon, 47 tRNA genes, and 9 CRISPR repeat sequences.

## ANNOUNCEMENT

The genus *Stygiolobus*, consisting of obligate anaerobic thermoacidophilic archaea, belongs to the order *Sulfolobales* (1) of the phylum *Crenarchaeota*. It is now represented by a single species, Stygiolobus azoricus (2). Recently, we isolated *Stygiolobus* sp. strain KN-1 under aerobic conditions. Although KN-1 and S. azoricus are phylogenetically very close (i.e., 16S rRNA gene sequence similarity of 98.9%), they are clearly different in terms of the ability to utilize molecular oxygen as an electron acceptor. Here, we report the complete genome sequence of strain KN-1.

Strain KN-1 was isolated by dilution to extinction from a hot spring water sample collected at the Unzen hot spring in Japan (65°C to 68.5°C, pH 2.3). Cultivation was conducted at 65°C using modified Brock's basal salt medium supplemented with yeast extract (MBSY) (pH 2.3) under aerobic conditions (3). To date, KN-1 has been found to grow both aerobically and anaerobically at growth temperatures between 55°C and 87.5°C (Table 1).

**TABLE 1:** Aerobic and anaerobic growth of strain KN-1 and Stygiolobus azoricus

Electron donor/acceptor	Growth	
	KN-1	Stygiolobus azoricus
S^0^/O_2_ (aerobic growth)^*a*^	+	−
H_2_/S^0^ (anaerobic growth)^*b*^	+	+

a The medium used for aerobic conditions was MBSY (pH 3.0) with S^0^ (1 g/liter) (headspace, air).

b The medium used for anaerobic conditions was MBSY (pH 3.0) with S^0^ (1 g/liter) (headspace, H_2_/CO_2_ [8:2]).

To obtain genomic DNA, 300 ml of the culture was centrifuged (10,000 × *g* at 4°C for 10 min). The DNA was extracted from the pellet using the Genomic-tip 100/G kit (Qiagen) and was used for DNA library preparation. We performed short- and long-read sequencing. For short-read sequencing, a DNA library was prepared with the NEBNext Ultra II FS DNA library preparation kit for Illumina (New England Biolabs). Short-read sequencing was conducted by Novogene Co., Ltd., on the Illumina NovaSeq 6000 platform (2 × 150 bp), resulting in a total of 6,435,458 raw reads (965,318,700 bp). The raw short reads were quality filtered using fastp v.0.20.1 (4), resulting in a total of 6,413,690 quality-filtered reads (912,689,516 bp). Long-read sequencing was conducted on a MinION sequencer with an R9 flow cell, SQK-LSK109, and EXP-NBD104 (Oxford Nanopore Technologies), following the protocol described by Oxford Nanopore Technologies (NBE_9065_v109_revZ_14Aug2019). DNA fragments of 3 kb or longer were enriched by the protocol. Base calling was performed by MinKNOW v.4.2.8 software (5). A total of 218,142 raw long reads (1,685,895,374 bp [*N*_50_, 11,988 bp]) were quality filtered using Filtlong v.0.2.0 (https://github.com/rrwick/Filtlong), resulting in a total of 141,549 quality-filtered long reads (1,000,018,187 bp [*N*_50_, 9,530 bp]). Using all of the quality-filtered reads, hybrid assembly was carried out with Unicycler v.0.4.8 (6). As a result, one circular contig composed of 2,958,410 bp was obtained (GC content, 40.1%). Finally, genome annotation was performed using DFAST v.1.4.0 (7). Default parameters were used for all software.

The genome of KN-1 contained 2,973 coding sequences, 1 copy of the 16S-23S rRNA operon, 47 tRNAs, and 9 CRISPR repeat regions. A BLAST search of full-length 16S rRNA sequences showed 98.9% sequence identity to S. azoricus. However, the pairwise average amino acid identity and average nucleotide identity values were 79.4% and 76.1%, respectively. Those values were below the species-level threshold (8, 9), indicating that KN-1 may be a novel species of the genus *Stygiolobus*.

Since strain KN-1 and S. azoricus are phylogenetically very close but different in terms of the ability to utilize molecular oxygen as an electron acceptor, the comparative genomic analysis of the two strains may reveal some key genes involved in anaerobic/aerobic respiration in the genus *Stygiolobus*.

### Data availability.

The complete genome sequence of strain KN-1 and the raw reads have been deposited in DDBJ/ENA/GenBank under the accession numbers AP024597, DRR286849, and DRR286850.
